# Phosphoinositides Play Differential Roles in Regulating Phototropin1- and Phototropin2-Mediated Chloroplast Movements in *Arabidopsis*

**DOI:** 10.1371/journal.pone.0055393

**Published:** 2013-02-06

**Authors:** Chhavi Aggarwal, Justyna Łabuz, Halina Gabryś

**Affiliations:** Department of Plant Biotechnology, Faculty of Biochemistry, Biophysics and Biotechnology, Jagiellonian University, Krakow, Poland; Instituto de Biología Molecular y Celular de Plantas, Spain

## Abstract

Phototropins are UVA/blue-light receptors involved in controlling the light-dependent physiological responses which serve to optimize the photosynthetic activity of plants and promote growth. The phototropin-induced phosphoinositide (PI) metabolism has been shown to be essential for stomatal opening and phototropism. However, the role of PIs in phototropin-induced chloroplast movements remains poorly understood. The aim of this work is to determine which PI species are involved in the control of chloroplast movements in *Arabidopsi*s and the nature of their involvement. We present the effects of the inactivation of phospholipase C (PLC), PI3-kinase (PI3K) and PI4-kinase (PI4K) on chloroplast relocations in *Arabidopsis*. The inhibition of the phosphatidylinositol 4,5-bisphospahte [PI(4,5)P2]-PLC pathway, using neomycin and U73122, suppressed the phot2-mediated chloroplast accumulation and avoidance responses, without affecting movement responses controlled by phot1. On the other hand, PI3K and PI4K activities are more restricted to phot1- and phot2-induced weak-light responses. The inactivation of PI3K and PI4K by wortmannin and LY294002 severely affected the weak blue-light-activated accumulation response but had little effect on the strong blue-light-activated avoidance response. The inhibitory effect observed with PI metabolism inhibitors is, at least partly, due to a disturbance in Ca^2+^_(c)_ signaling. Using the transgenic aequorin system, we show that the application of these inhibitors suppresses the blue-light-induced transient Ca^2+^_(c)_ rise. These results demonstrate the importance of PIs in chloroplast movements, with the PI(4,5)P2-PLC pathway involved in phot2 signaling while PI3K and PI4K are required for the phot1- and phot2-induced accumulation response. Our results suggest that these PIs modulate cytosolic Ca^2+^ signaling during movements.

## Introduction

The UV-A/blue-light (BL) photoreceptor phototropin is involved in regulating light dependent processes that are important for the promotion of plant growth and for the optimization of photosynthetic efficiency [1]. Phototropins use flavin mononucleotides as chromophores and become auto-phosphorylated upon BL irradiation by the serine / threonine kinase domain at the C-terminal [2]. Although identified in various plant species, *Arabidopsis thaliana* has served as a basic model organism to study phototropin-mediated effects. *Arabidopsis* contains two phototropins, phot1 and phot2, which regulate various responses, including phototropism, stomatal movements, chloroplast redistribution, hypocotyl elongation inhibition and leaf positioning [1]. Phototropin-induced chloroplast movements are important for optimizing photosynthetic activity [3]. Under weak BL, phot1 and phot2 mediate a chloroplast accumulation response in which chloroplasts move to the periclinal cell walls (face position) in order to capture more light for photosynthesis [4]. In strong BL, chloroplasts move away from the site of irradiation to the anticlinal walls (profile position) to prevent photo-damage of the photosynthetic apparatus [5], [6]. The avoidance response of chloroplasts is mediated solely by phot2 [7].

The rapid turnover of membrane lipids, in particular phoshoinositides (PIs), modulates a wide range of cellular processes. One of the earlier concepts of signal transduction utilizing PI involves the PI-PLC (phospholipase C) pathway which is widely known for the release of Ca^2+^ from intracellular compartments. The PLC is triggered by cell membrane initiated signaling pathways and results in the production of diacylglycerol (DAG) and the calcium-mobilizing messenger inositol 1,4,5-triphosphate [Ins(1,4,5)P3] from phosphatidylinositol 4,5-bisphosphate [PI(4,5)P2] hydrolysis [8]. The importance of intracellular Ca^2+^ stores in BL-activated chloroplast movements has been shown in *Lemna trisulca* [9], [10]. In *Lemna*, inhibitors which disturb Ca^2+^ levels in internal stores (caffeine and thapsigargin) suppressed the movements within a few minutes of application. Additionally, Harada et al. [11] proposed that phot2 mediates Ca^2+^ influx both from the apoplast via plasma membrane Ca^2+^ channels and from the internal stores via the activation of PLC. Today the PI-PLC pathway is known to be involved in diverse processes including stomatal movement [12], pollen tube and root growth [13], [14], disease resistance [15] and abiotic stress [16].

In recent years, apart from PI(4,5)P2, other PI species have also been identified as important signaling molecules, particularly phosphatidylinositol 3-phosphate (PI3P) and 4-phosphate (PI4P). Phosphatidylinositol 3-kinase (PI3K) and PI4K phosphorylate specific positions in the inositol ring of PI and produce PI3P and PI4P respectively. PI3P is present in a very low content in plant cells. *Arabidopsis* contains a single AtPI3K gene. PI3P has been implicated in various events, including endocytosis, ROS production during defense responses and guard cell closure by ABA [17]. The other phosphorylated PI, PI4P, accounts for about 80% of total plant PIs and a family of 12 PI4Ks has been predicted in *Arabidopsis*. Apart from functioning as a precursor of PI(4,5)P2, PI4P has also been suggested to be a direct substrate for PLC [18]. In plants, the importance of PI4P has been reported in cell division, root hair and pollen tube growth and stomatal movements [17]. In *Lemna trisulca* and *Nicotiana tabacum* involvement of these phosphorylated PIs has been proposed during chloroplast movement responses. Wortmannin (WM) specifically inhibits PI3K at low concentrations and both PI3K and PI4K at higher concentrations. Studies on *L. trisulca* demonstrated that chloroplast accumulation and avoidance movements are differently sensitive to this inhibitor [19]. The authors suggested a major role for PI3K in the chloroplast accumulation response under weak BL, the mechanism of its action remained although unclear. Similar results were also reported by Anielska-Mazur et al. [20] for *N. tabacum*. Interestingly, the exogenous application of Ca^2+^ partially restored movements in WM treated *Nicotiana* leaves.

Changes in the PI metabolism have been shown to be important for other phototropin-mediated processes involving phototropic responses and guard cell movements. The expression of inositol polyphosphate 5-phosphatase13 (Ins5Ptase13) is suppressed by phot1 and this results in shortened hypocotyl [21]. Furthermore, the constitutive expression of human type I Ins5Ptase in *Arabidopsis* leads to reduced hypocotyl bending after directional BL exposure [22]. Ins5Ptases are crucial for dephosphorylating Ins(1,4,5)P3, Ins(1,3,4,5)P4 and PI(4,5)P2, and thus manage cytosolic Ca^2+^ mobilization [23]. In *Arabidopsis* guard cells, illumination with white light elevates PI(4,5)P2 content thereby inactivating an anion channel and resulting in stomatal opening [12]. Recent work showed BL inhibition of anion current from guard cells in *Arabidopsis* and *Vicia faba* [24]. In general, PIs perform different functions in cells depending on the tissue.

The aim of this work is to find the PI species involved in regulating phototropin-controlled chloroplast relocations and their mechanism of action. We used a pharmacological approach to investigate the involvement of PIs in the control of chloroplast movements in *Arabidopsi*s. We report that phot2, not phot1, activates the PI(4,5)P2-PLC pathway upon perceiving BL, and this plays an important role in phot2 signaling during movement responses. On the other hand, PI3P and PI4P are more specifically involved in the phot1- and phot2-mediated accumulation response and modulate the Ca^2+^_(c)_ levels.

## Materials and Methods

### Plant Material and Inhibitor Treatments

*Arabidopsis thaliana* wild type (WT) Columbia seeds were obtained from Nottingham *Arabidopsis* Stock Center (Nottingham, UK). The seeds of phototropin mutants were the kind gift of Anthony R. Cashmore, Plant Science Institute, Department of Biology, University of Pennsylvania, Philadelphia, USA (*phot1, phot2*). The *Arabidopsis* transgenic line expressing cytosolic aequorin was obtained from the lab of Marc R. Knight, Plant Stress Laboratory, School of Biological and Biomedical Sciences, Durham University, UK. Seeds (WT, *phot1*, *phot2* and the transgenic line expressing cytosolic aequorin) were sown on Jiffy-7 pots (Jiffy products international AS) and grown in a growth chamber (Sanyo MLR 350H, Japan) at 23°C, PPFD of 70–100 µmol m^−2^ s^−1^ and 80% relative humidity. A photoperiod of 10 h light/14 h dark was maintained to prevent early flowering of plants and to develop mature leaves. All experiments were performed on fully grown leaves of 4–6 week old *Arabidopsis* plants.

Inhibitor treatments: Both WT and mutant plants of *Arabidopsis* were dark-adapted for at least 12 h. Mature leaves were detached and infiltrated with either neomycin (BioShop), U73122 (Merck), U73343 (Merck), wortmannin (Sigma) or LY294002 (Sigma). All working solutions were buffered with 10 mM PIPES (pH 6.8, Sigma). Photometric and microscopic studies were performed after incubating the infiltrated leaves for 60 min (neomycin, U73343 and U73122) or 90 min (wortmannin and LY294002) in darkness, with slow stirring. All manipulations were done under safe green light.

### Determination of PI(4,5)P2 and PI3P Content

The effects of neomycin and U73122 on the PI(4,5)P2 metabolism, and of WM and LY294002 on PI3P formation were tested using K4500: PI(4,5)P2 mass Elisa kit and K3300: PI3P mass Elisa kit respectively (Echelon bioscience). The assay was performed according to the manufacturer’s protocol. In brief, *Arabidopsis* leaves were infiltrated with either 250 µM neomycin, 25 µM U73122, 10 µM WM, 200 µM LY294002 or 10 mM PIPES (pH 6.8). After 60/90 min incubation leaves were either kept in darkness or illuminated with BL (2 µmol m^−2 ^s^−1^) for 60 min. Low intensity BL was chosen for illumination because all the inhibitors (neomycin, U73122, WM and LY294002) suppressed the weak BL movement responses (results section). The samples were frozen immediately in liquid nitrogen. The leaves were ground in a mortar and pestle using liquid nitrogen and weighed. The acidic lipids (containing PIs) were extracted using CHCl_3_, CH_3_OH and HCl. The extracted PI(4,5)P2/PI3P were first incubated with a PI(4,5)P2/PI3P detector and then added to a PI(4,5)P2/PI3P coated microplate for competitive binding. A peroxidase linked secondary detector and colorimetric substrate were then used to detect the PI(4,5)P2/PI3P detector protein bound to the plate. The colorimetric signal was inversely proportional to the amount of PI(4,5)P2/PI3P extracted from cells. The concentrations of PI(4,5)P2 and PI3P were calculated from standard curves.

### Photometric Method

**Figure 1:**
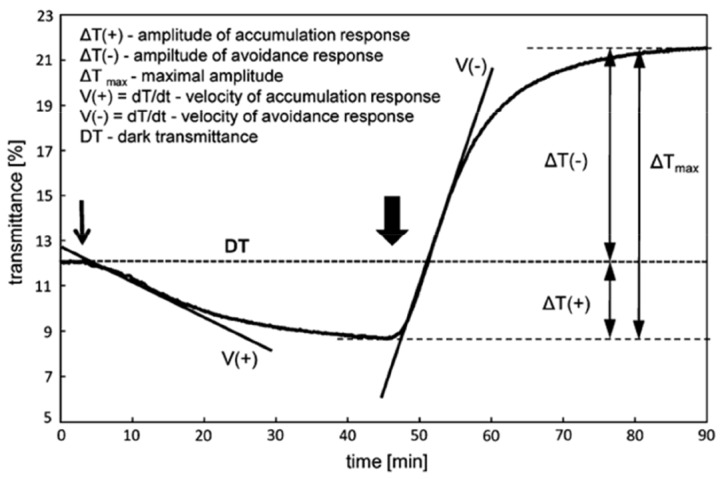
Chloroplast movements in *Arabidopsis* in response to continuous BL. Photometer model curves showing changes in transmittance (ΔT) of red measuring light through dark-adapted leaves exposed to continuous weak BL (1.6 µmol m^−2 ^s^−1^, 45 min, the onset marked with a thin arrow) and strong BL (120 µmol m^−2 ^s^−1^, 45 min, the onset marked with a thick arrow). Parameters for chloroplast responses: amplitude and velocities are shown the way they are measured / calculated.

Quantitative measurements of chloroplast movements were performed using a double-beam photometer [25] which records changes in red light transmittance (660 nm, 0.1 µmol m^−2 ^s^−1^, modulated with a frequency of 800 Hz) through the leaf. After measuring the initial level of transmittance, leaves were illuminated with weak BL (1.6 µmol m^−2 ^s^−1^) for 45 min, followed by strong BL (120 µmol m^−2 ^s^−1^) for the same time period. The following parameters were measured / calculated for each response: (1) the amplitude of transmittance changes after 45 min, and (2) the velocity - the first derivative of the initial linear fragment of the transmittance curve. An example of movement responses and the calculated parameters are shown in **Fig. 1**. The BL was obtained from light emitting diodes with an emission maximum at 460 nm (Philips Lumiled Lighting Company, San Jose, California, USA).

### Microscopic Studies

*Arabidopsis* WT plants were dark-adapted for 12 h. The leaves were detached, the lower epidermis was removed and the tissue was cut into small pieces (≈20 mm^2^). The leaf pieces were kept in a solution with 10 mM PIPES or neomycin (10, 100 or 250 µM) for 60 min. The chloroplast positioning in the mesophyll cells was then photographed at 40X magnification (Nikon eclipse TE200) before BL-illumination (at 0 min), after strong BL-illumination (100 µmol m^−2^ s^−1^ for 60 min) and after weak BL-illumination (2 µmol m^−2^ s^−1^ for 60 min) using ProPlus software. BL irradiation was performed in the microscope using a glass filter with the emission maximum at 460±10 nm. Fluence rates were measured with a silicon photodiode calibrated against a LI-COR quantum meter (Li-Cor, Lincoln, NB, USA).

### Aequorin Studies

Aequorin luminescence was measured using a GlowMax 20/20 luminometer (Promega). Leaf discs from transgenic *Arabidopsis* plants (expressing cytosolic aequorin, a calcium-sensitive luminescent protein) were dark-incubated for 10–12 h with a freshly prepared 2.5 µM coelenterazine (p.j.k) solution in 1.5 ml eppendorf tubes. After incubation, the tubes were placed in the tube holder of the luminometer, under complete darkness. The basal luminescence was recorded for 5 min. Then the discs were irradiated with BL (100 µmol m^−2 ^s^−1^) for 5 s and the light emitted from the discs was recorded. BL was obtained from a photodiode of 457±10 nm wavelength. For the inhibitor treatments U73122, U73343, wortmannin or LY294002 were added 60 min before the luminescence measurements. At the end of the experiment the remaining reconstituted aequorin was discharged by adding 1 M CaCl_2_ in 10% ethanol. The luminescence counts obtained with the luminometer were calibrated into Ca^2+^ concentrations using the equation of Rentel and Knight [26].

## Results

### PI(4,5)P2-PLC Inhibitors Suppress Chloroplast Movements in *phot1* but not in *phot2* Mutant Plants

In order to determine the involvement of PI(4,5)P2 in the regulation of chloroplast movements, leaves of *Arabidopsis thaliana* were treated with increasing doses of neomycin or U73122. Neomycin is a poly-cationic aminoglycoside antibiotic which binds to PI(4,5)P2 and thereby prevents its hydrolysis by PLC [27]. U73122 is an aminosteroid which has been extensively used as a pharmacological inhibitor of the PI-PLCs in plants [28].

**Figure 2:**
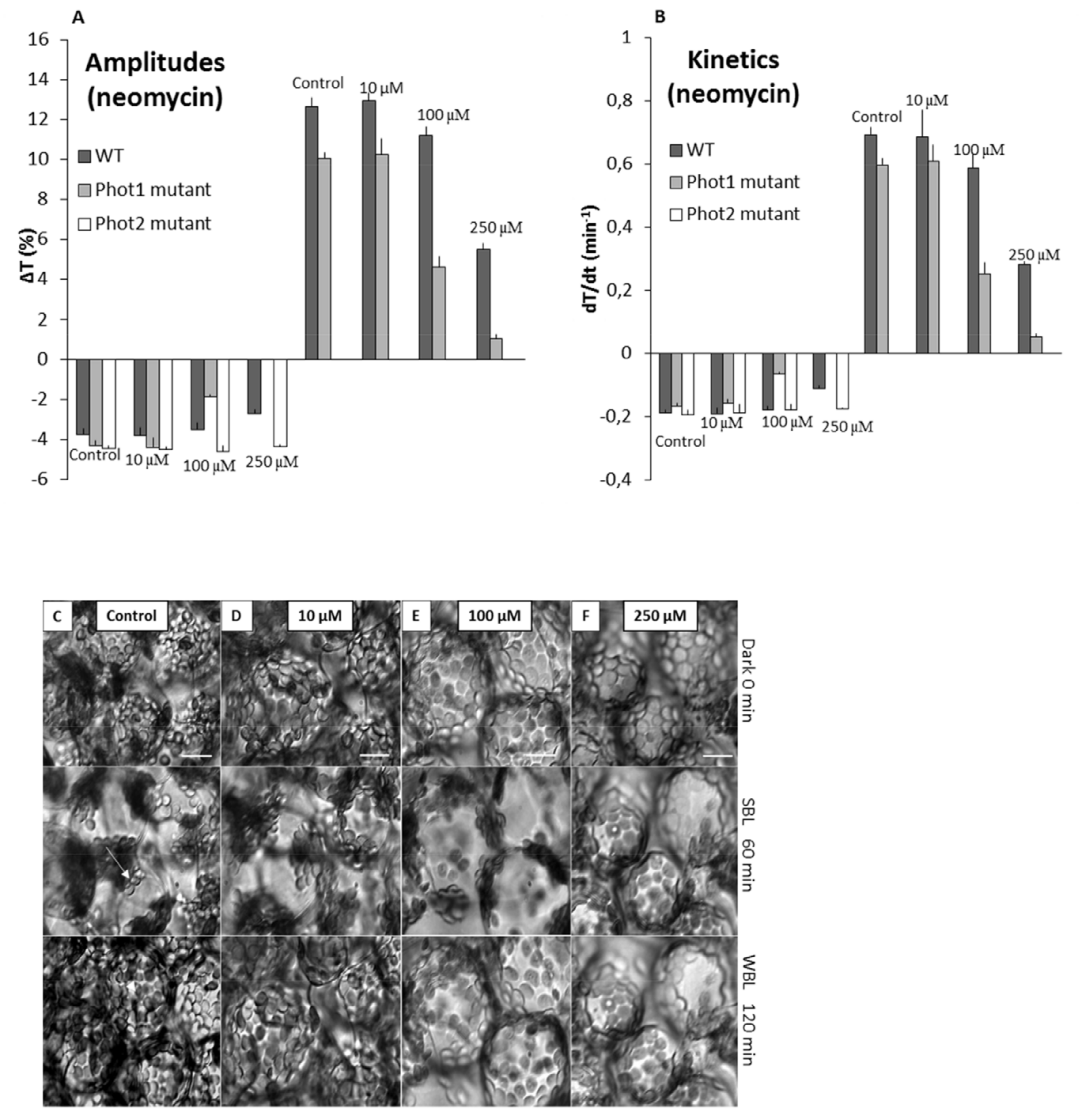
Chloroplast responses to BL in *Arabidopsis* leaves pretreated with neomycin. A and B Parameters of chloroplast responses in WT plants and *phot1* mutant after application of 10 mM PIPES, 10, 100 or 250 µM neomycin: (A) amplitudes and (B) kinetics. Data values are mean ± SE (n = 10) from 3 independent experiments. The minus values on the graphs represent the parameters for weak BL response (1.6 µmol m^−2 ^s^−1^) and the plus values indicate the strong BL response parameters (120 µmol m^−2 ^s^−1^). **C, D, E and F** The chloroplast positioning in the mesophyll cell layer. Dark-acclimated *Arabidopsis* WT leaf tissues were irradiated with strong BL (100 µmol m^−2 ^s^−1^ for 60 min) followed by weak BL (2 µmol m^−2 ^s^−1^ for 60 min) in the (C) absence or (D, E, F) presence of neomycin (10, 100, 250 µM). Images from time point 0 min, 60 min and 120 min are shown, taken at 40X. Arrow show chloroplasts at anticlinal cell wall and arrowhead show chloroplasts at periclinal cell wall. Bar = 20 µm.

At 10 µM neomycin, accumulation and avoidance responses were unaffected in WT, *phot1* and *phot2* mutants. Both amplitudes and kinetics (as depicted in **Fig. 1**) were comparable to control samples (10 mM PIPES) (**Fig. 2A, 2B**). Upon increasing the neomycin concentration to 100 µM, the parameters of chloroplast responses (amplitude and velocity) were reduced by about 10% in the WT plants, the decrease being more prominent in the avoidance response (**Fig. 2A, 2B**). At the same concentration the inhibitory effect was remarkably stronger in the *phot1* mutant, with more than 50% suppression of chloroplast responses (**Fig. 2A, 2B**). Movements in the *phot2* mutant were not suppressed at 100 µM neomycin (**Fig. 2A, 2B**). Subsequently, 250 µM neomycin abolished movements almost completely in the *phot1* mutant, with no chloroplast relocation under weak BL, and only 5–10% of the movement response under strong BL (**Fig. 2A, 2B**). In WT plants, the inhibitory effect was not as strong as in the *phot1* mutant. The movement parameters were reduced by about 50% and the inhibition was again stronger in the avoidance response than in the accumulation (**Fig. 2A, 2B**). Even at 250 µM neomycin no changes were seen with respect to movements in the *phot2* mutant. To verify the inhibitory effect of neomycin on chloroplast movements, inverted microscopy was used to monitor the distribution of chloroplasts in the mesophyll cells of WT *Arabidopsis*. The microscopy data confirmed the neomycin effect on movement responses and was consistent with the results measured photometrically. At 10 µM neomycin, movements (both accumulation and avoidance) in mesophyll cells were similar to those in controls (10 mM PIPES) (**Fig. 2C, 2D**). However, an increase in concentration to 100 µM exhibited an inhibitory effect on movements (**Fig. 2E**). The analysis of chloroplast positions showed that only 50±5% of the chloroplasts were located at the periclinal (after weak BL) or anticlinal (after strong BL) cell walls (**Fig. 2E**). Finally, the application of 250 µM neomycin completely stopped the redistribution (**Fig. 2F**).

**Figure 3:**
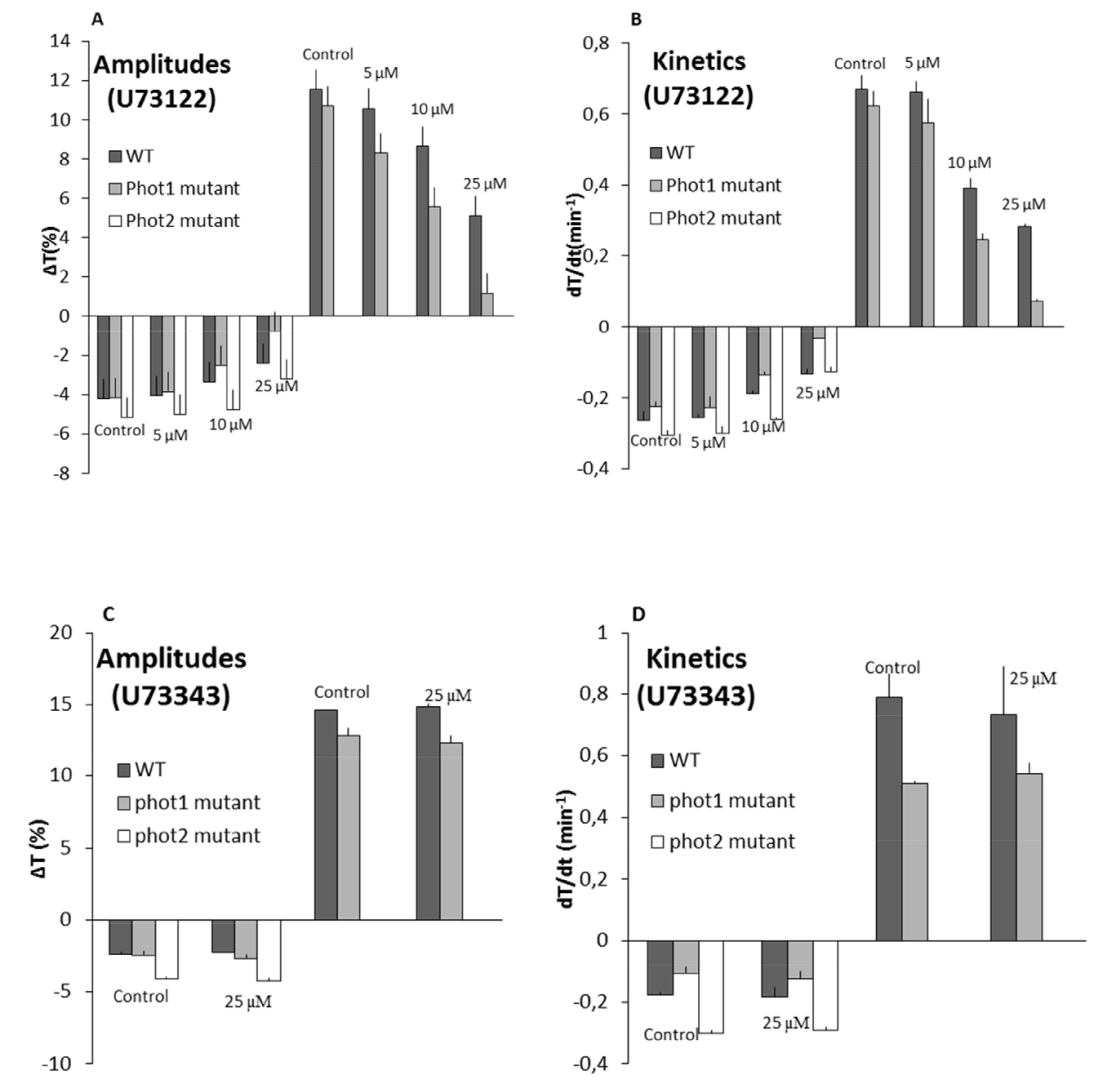
Influence of U73122 and U73343 on chloroplast movements in WT plants and ***phot*** mutants. A and B Data from leaves infiltrated with U73122 (0, 5, 10, 25 µM) and incubated in darkness for 1 h. Parameters of chloroplast responses: (A) amplitudes and (B) kinetics. **C and D** Chloroplast movement parameters in leaves treated with 10 mM PIPES or 25 µM U73343, (C) amplitudes and (D) kinetics. Data values are mean ± SE (n = 10) from 2 independent experiments.

With the application of the second inhibitor, U73122, at 5 µM a small decrease in the parameters of chloroplast avoidance response was observed in the *phot1* mutant (20% decrease in amplitude and 10% in kinetics). Insignificant inhibitory effects were seen in the WT and no changes were observed in the *phot2* mutant (**Fig. 3A, 3B**). Upon increasing the concentration to 10 µM a two-fold decrease in the *phot1* mutant and a 20% decrease in WT were seen in the parameters of chloroplast responses. The chloroplast movements in the *phot2* mutant were similar to control samples (**Fig. 3A, 3B**). A final concentration of 25 µM almost completely inhibited both accumulation and avoidance responses in the *phot1* and also reduced the movement parameters to 50% in the WT. In addition, at this concentration a small decrease in both amplitudes and kinetics was also observed in the *phot2* mutant (**Fig. 3A, 3B**). To confirm the inhibitory effects observed with U73122, its inactive structural analog U73343 was used as a negative control. Leaves treated with 25 µM U73343 showed movements similar to the controls (10 mM PIPES) in WT, *phot1* and *phot2* plants (**Fig. 3C, 3D**).

**Figure 4:**
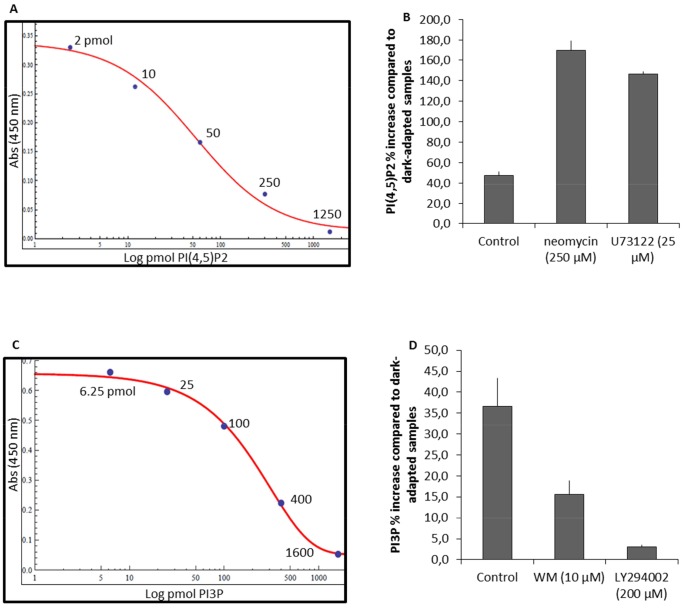
Effects of inhibitors on PI levels in leaves irradiated with 2 µmol m^−**2 **^s^−1^ of BL. A and C Standard plot of absorbance at 450 nm versus (A) log pmol PI(4,5)P2 and (C) log pmol PI3P. **B** Percentage change in PI(4,5)P2 content after BL irradiation in control (10 mM PIPES), neomycin (250 µM) and U73122 (25 µM) samples. **D** Percentage change in PI3P level after BL irradiation in control (10 mM PIPES), WM (10 µM) and LY294002 (200 µM). The vertical bars represent SE of 3 independent experiments.

We checked the inhibitory action of the two drugs (neomycin and U73122) towards the PI-PLC pathway. The PI(4,5)P2 levels were analyzed in *Arabidopsis* leaves after BL irradiation using an Echelon Bioscience mass Elisa kit (K4500). From the known PI(4,5)P2 concentration (as provided by the manufacturer), a sigmoidal graph was plotted of absorbance (450 nm) versus log pmol PI(4,5)P2 (**Fig. 4A**). *Arabidopsis* WT leaves irradiated with BL (2 µmol m^−2 ^s^−1^) showed a 45% increase in the PI(4,5)P2 amount relative to the dark-adapted samples. In comparison to the BL-irradiated control samples, treatments with 250 µM neomycin or 25 µM U73122 resulted in higher PI(4,5)P2 contents (170% and 145% respectively) after BL exposure (**Fig. 4B**).

### WM and LY294002 Inhibited Chloroplast Accumulation Response

In order to find out whether other PIs play a role during phot1 signaling, we decided to investigate whether PI3P and PI4P regulate chloroplast movements in *Arabidopsis* using WM and LY294002. The two drugs when used in the micromolar range suppress the activity of PI3K and PI4K, with LY294002 more specific for PI3K [29], [30].

**Figure 5:**
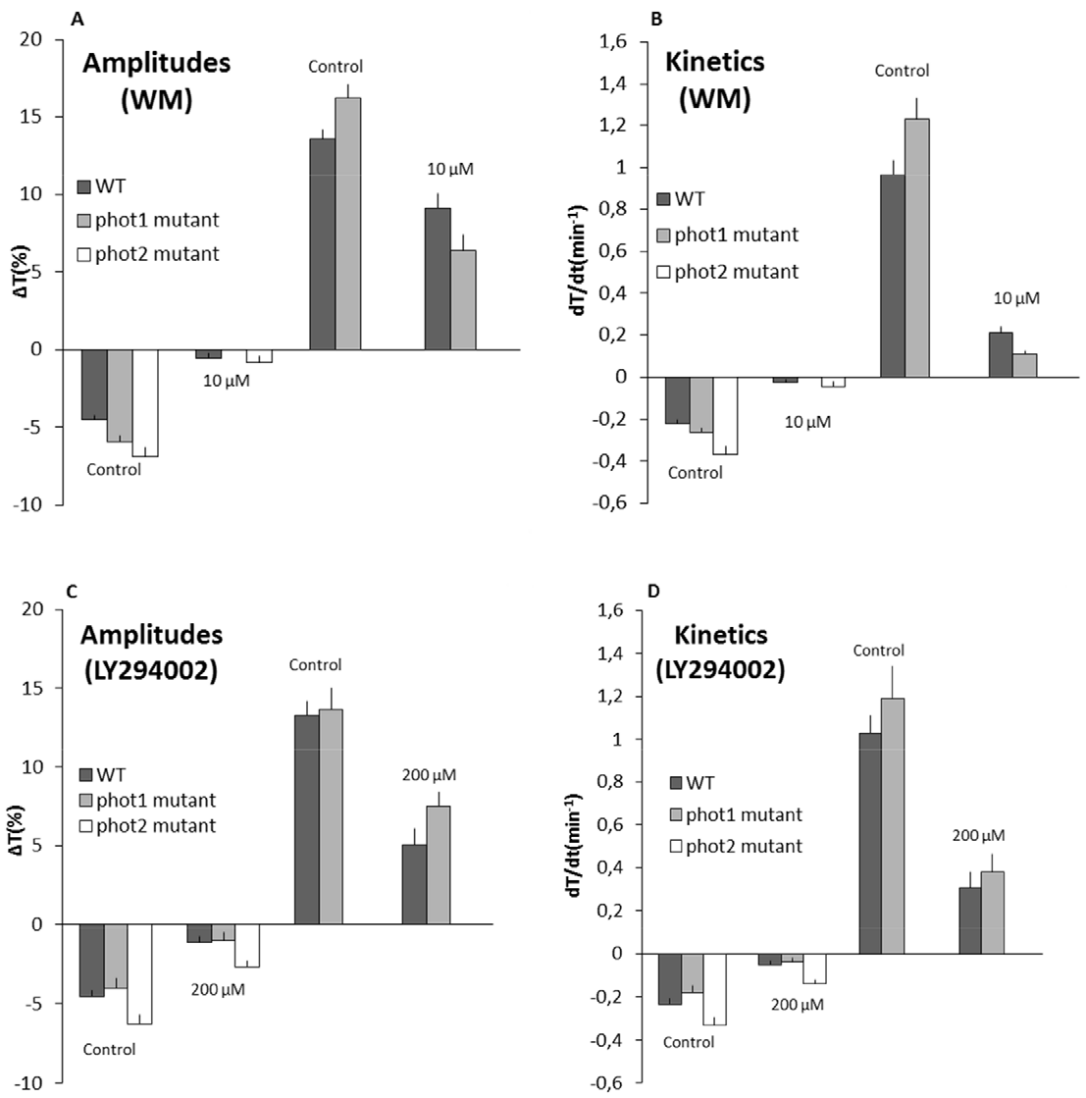
Effect of wortmannin (WM) and LY294002 on chloroplast movements in *Arabidopsis*. Leaves were infiltrated with (**A and B**) WM (10 µM) or (**C and D**) LY294002 (200 µM) and incubated for 1.5 h under darkness. Parameters of chloroplast responses: (A, C) amplitudes and (B, D) kinetics after treatment with inhibitors. Final readings are presented as mean ± SE (n = 10) from 2 independent experiments.

At 10 µM WM, a significant inhibition of the accumulation response was seen in WT, *phot1* and *phot2* mutants. The weak BL response was completely eliminated in the *phot1* mutant and the amplitudes were decreased ten-fold in WT and the *phot2* mutant plants. On the other hand, WM reduced the amplitudes of the avoidance response in WT by 30% and in the *phot1* mutant by 50% (**Fig. 5A**). Likewise, LY294002 affected the accumulation response more severely than the avoidance response. At 200 µM LY294002, the amplitudes of chloroplast movements under weak BL remained at 20% (WT and *phot1* mutant) and 40% (*phot2* mutant) of the control. In contrast, at the same concentration the amplitude of avoidance response remained at about 50% (WT and the *phot1* mutant) of the control (**Fig. 5C**). The influence of both inhibitors was stronger on kinetics than on amplitudes in both weak BL and strong BL responses (**Fig. 5B, 5D**).

In a manner similar to PI(4,5)P2, the effects of WM and LY294002 on PI3K were also tested using the Echelon Bioscience mass Elisa kit (K3300). A standard sigmoidal curve was plotted for absorbance (450 nm) versus log pmol PI3P (**Fig. 4C**). *Arabidopsis* leaves illuminated with BL (2 µmol m^−2 ^s^−1^) showed a 36% increase in the PI3P amount compared to dark-adapted samples. Pretreatment of leaves with 10 µM WM or 200 µM LY294002 resulted in only a 15% and 3% rise respectively after BL irradiation (**Fig. 4D**).

### Phosphoinositide Inhibitors Suppress BL-induced Transient Rise of Ca^2+^_(c)_ in *Arabidopsis* Leaves

Phototropin-induced chloroplast movements are associated with changes in Ca^2+^_(c)_ levels. PI(4,5)P2, PI3P and PI4P are known to be involved in mobilizing calcium to the cytoplasm from internal stores and/or plasma membrane Ca^2+^ channels. So we examined whether these PI species are associated with BL-mediated Ca^2+^ signaling in *Arabidopsis* leaves, using a transgenic *Arabidopsis* WT line expressing cytosolic aequorin.

**Figure 6:**
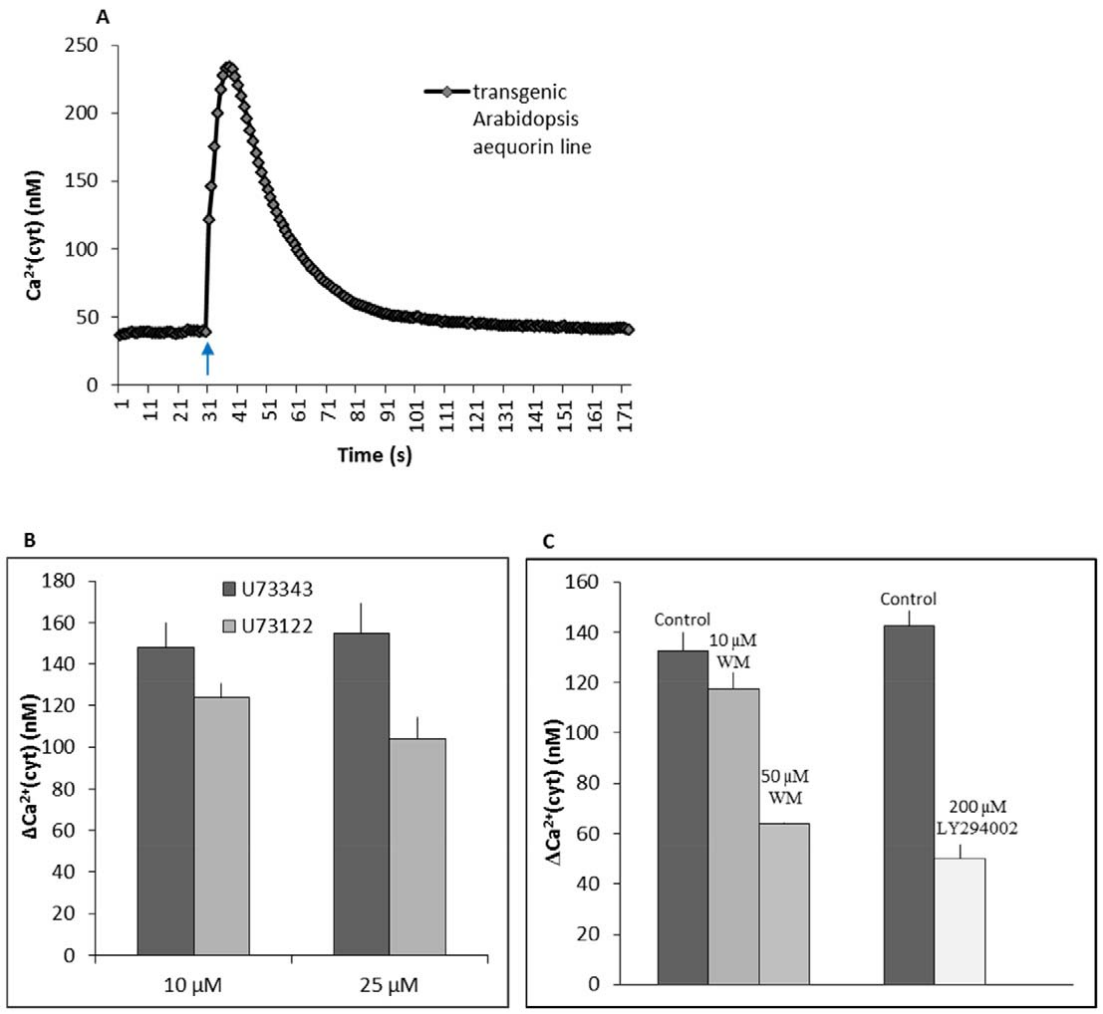
Effects of inhibitors on BL-induced Ca^2+^_(c)_ in the WT transgenic plants. **A** Leaf discs were illuminated with 100 µmol m^−2^ s^−1^ of BL for 5 s. **B and C** Leaf discs treated with (B) 10/25 µM U73122 or U73343 and (C) 10 µM WM, 50 µM WM or 200 µM LY294002 60 min before the measurements and then irradiated with 100 µmol m^−2 ^s^−1 ^BL for 5 s. The vertical bars represent SE of 20 independent experiments for each. The arrow shows the onset of BL pulse.

Leaf discs from WT plants showed a transient rise in Ca^2+^_(c)_ when irradiated with 100 µmol m^−2 ^s^−1^ BL for 5 s (**Fig. 6A**). The rise attained a peak after 10 s and lasted about 60 s. The resting level of Ca^2+^_(c)_ in the dark was 40±5 nM and the peak Ca^2+^_(c)_ after BL induction was 220±20 nM. We examined the changes in Ca^2+^_(c)_ in the presence of PI-PLC, PI3K and PI4K inhibitors. Leaf discs treated with 10 µM U73122 showed a 15% decrease in the peak Ca^2+^_(c)_ value as compared to leaves with 10 µM U73343, the inactive analog. When the concentration was increased to 25 µM the transient rise was reduced by 30% of the control values (25 µM U73343) (**Fig. 6B**). Simultaneously, a two-fold increase in dark levels of Ca^2+^_(c)_ was observed in the presence of 25 µM U73122 (**Fig. S1**). WM treatment showed no changes in dark Ca^2+^_(c)_ levels. However, LY294002 showed a small increase in the dark levels of Ca^2+^_(c)_ (**Fig. S1**). Pretreatment with WM or LY294002 resulted in a prominent decrease in BL-induced transient Ca^2+^_(c)_ rise. At 10 µM WM the BL-induced Ca^2+^_(c)_ level was reduced by 10% of the control leaves (0.5% DMSO in water). An increase in concentration to 50 µM suppressed the mean peak value to 60±10 nM compared to the 130±5 nM observed with control samples. Similarly, pretreatment with 200 µM LY294002 inhibited the transient by 65% of control leaves (**Fig. 6C**).

## Discussion

The involvement of PIs has always been suggested in BL-induced chloroplast movements [9], [10], [19], [20], but has never been addressed in detail. Our attempt here is to identify the PI species involved in signaling during the movement responses and to assess their mode of action.

### PI(4,5)P2-PLC Pathway is Involved in Phot2-mediated Movements

In plants PI-PLC activation and the resultant Ca^2+^ release initiate a variety of enzymatic and structural changes in downstream signaling elements. The PI(4,5)P2 binding agent, neomycin and the PLC inhibitor, U73122, both suppressed PI(4,5)P2 turnover in the BL irradiated *Arabidopsis* leaves (**Fig. 4B**). Treatments with neomycin or U73122 inhibited phototropin-mediated chloroplast movements in a dose-dependent manner which was observed by photometry and microscopy (**Fig. 2** **,** **3**). The suppressive effect of neomycin was seen only in WT and *phot1* plants. The movement response in the *phot2* mutant (where only phot1 is active) was not affected by neomycin even at higher concentrations (**Fig. 2**). This indicates that PI(4,5)P2 is not a downstream component in the phot1-initiated signal transduction process during chloroplast movements. In a similar manner, U73122 inhibited chloroplast movements in *phot1* mutant and WT. However, at a high micromolar concentration U73122 was effective in a slight suppression of movements in *phot2* plants (**Fig. 3**). This difference between neomycin and U73122 can be explained because of the different target sites for the two drugs. In contrast to neomycin, U73122 directly inhibits PLC, and both PI(4,5)P2 and PI4P have been shown to be substrates for PLC [18]. Taking into account the null effect of neomycin, we suggest that the inhibition observed in the *phot2* mutant after U73122 application was due to a disturbance of the PI4P turnover. This conclusion was further supported by WM studies (next section).

### PI-PLC Activation Induces Cytosolic Ca^2+^ Increase upon BL Irradiation

How does the PI-PLC pathway work in phototropin signaling? The substrate for PLC, PI(4,5)P2, is involved in numerous processes in the cell, including Ca^2+^ signaling [14], membrane trafficking [31], actin cytoskeleton arrangement [13], [14], ion channel activity [12]. Actin and Ca^2+^ signaling are both known to be crucial for chloroplast movements in response to BL [20], [32], [33]. The staining of F-actin in the mesophyll cells of *N. tabacum* after treatment with neomycin (100 µM) showed no detectable effect on the actin network arrangement under dark conditions or after BL-illumination as compared to control samples (**Fig. S2**). However, we cannot eliminate the possibility that transgenic lines with GFP fused to actin binding proteins might provide a more clear answer. To study Ca^2+^ signaling in the presence of BL we used a transgenic *Arabidopsis* WT line (expressing cytosolic aequorin). Leaves from the transgenic line showed a transient rise of cytosolic Ca^2+^ upon pulse irradiation with BL (5 s, 100 µmol m^−2 ^s^−1^) (**Fig. 6A**). This rapid transient rise upon BL illumination has also been reported in different tissues of *Arabidopsis*: hypocotyl [34], leaves [11], guard cells and mesophyll cells [35]. Inactivation of the PI-PLC pathway by U73122 disturbs the Ca^2+^ efflux from intracellular organelles [36]. The application of U73122 suppressed the BL-induced transient rise of Ca^2+^_(c)_ in transgenic *Arabidopsis* WT leaves (**Fig. 6B**). This points to the importance of Ca^2+^ release from internal stores upon phototropin activation. Indeed, Harada et al. [11] reported that pretreatment with neomycin or U73122 suppresses the BL-induced (0.1 µmol m^−2 ^s^−1^, 10 s pulse) Ca^2+^_(c)_ increase in transgenic *Arabidopsis* WT and in *phot1* mutant plants expressing cytosolic aequorin. We obtained results similar to U73122 with the application of neomycin (data not shown); however, since the transgenic aequorin *Arabidopsis* plants contained neomycin phosphotransferase II gene, the data obtained with neomycin was not considered for the study. The results obtained in our studies confirm the data of Harada et al. [11], and ascertain that the activation of the PLC pathway by phot2 and the thereby activated Ca^2+^ release from internal stores is important for the directional movement responses of chloroplasts, while phot1 works differently. Intriguingly, the inhibitory effects of neomycin and U73122 observed using photometry were always stronger in the *phot1* mutant than in WT plants (**Fig. 2** **,** **3**). This suggests the potential participation of both phototropins in mediating cytosolic calcium changes for optimal chloroplast movements. Various earlier studies have reported that the activation of phot1 allows a Ca^2+^ influx from the apoplast [11], [34], [37], [38]. Additionally, phot1 suppresses inositol polyphosphate 5-phosphatase activity [hydrolyzes the phosphate group at the 5′position of Ins(1,4,5)P3] which results in enhanced cytosolic Ca^2+^ and thus leads to reduced hypocotyl length [21].

### PI3P and PI4P are Involved During Phototropin-mediated Accumulation Response

To address the question of whether other PI species are involved in the phot1-mediated accumulation response, we assessed the significance of PI3P and PI4P during chloroplast movements. The specific PI3K inhibitors WM (a fungal metabolite) and LY294002 (a synthetic molecule) have been invaluable tools in elucidating the roles of these enzymes in signal transduction pathways. WM has been used previously and was reported to inhibit the BL-induced movement responses in *Lemna trisulca* and *Nicotiana tabacum*, with lower concentrations being needed to inhibit the accumulation response [19], [20]. An increase in the PI3P level was observed upon irradiation of *Arabidopsis* WT leaves with low intensity BL. Treatment of the leaves with WM or LY294002 suppressed this increase (**Fig. 4D**), showing the specific effect of these drugs on PI3K enzyme activity. In the present study, WM and LY294002 inhibited the phot1- and phot2-mediated chloroplast accumulation response severely, while only partially affecting the avoidance response. These results indicate the importance of PI3P and PI4P for chloroplast positioning to the periclinal walls under weak BL. In contrast, these phosphorylated PIs have only a minor role during the anticlinal positioning of chloroplasts. Interestingly, PI3K and PI4K activities have also been shown in the spinach chloroplast outer envelope membrane. Furthermore, the presence of WM inhibits PI phosphorylation in the chloroplast envelope [39]. It would be interesting to examine PI kinase activities at the chloroplast envelope in *Arabidopsis* phototropin mutants in the absence/presence of BL.

A stronger decrease in amplitude was seen with WM under weak BL in *phot1* and *phot2* mutants as compared with LY294002 (**Fig. 5A, 5B**). This difference may be due to the difference in the drugs used (WM and LY294002). LY294002 specifically inhibits PI3K while WM inhibits not only PI3K but also PI4K **[29]**, **[30]**.

### PI3K and PI4K Inhibition Disturbs BL-induced Cytosolic Ca^2+^ in *Arabidopsis* Leaves

Reports from both plant and animal models show that cytosolic Ca^2+^ increases via the PI3K and PI4K pathways. PI3P and PI4P are involved in ABA-induced stomatal closure through an increase in cytosolic Ca^2+^ [40]. Phosphatidylinositol 3,5-bisphosphate (synthesized from PI3P) modulates intracellular Ca^2+^ by activating ryanodine receptors in smooth muscle cells [41]. Additionally, PI4P acts as a direct substrate for PLC in tomato suspension cells where it triggers an oxidative burst [18]. The exogenous application of Ca^2+^ negated the inhibitory effect observed with WM on chloroplast movement responses in *N. tabacum* [20]. In our studies, the pretreatment of aequorin-expressing *Arabidopsis* WT leaves with WM or LY294002 substantially reduced the BL-mediated transient rise in Ca^2+^_(c)_, indicating that phototropin-induced Ca^2+^ release is affected, but not eliminated entirely (**Fig. 6C**). In plant cells the source of Ca^2+^ mobilization via PI3P and PI4P is not known. However, the results obtained in this work show that phosphorylation of PI via PI3K and PI4K modulate cytosolic Ca^2+^ levels upon phototropin activation. Apart from Ca^2+^ signaling, PI3K and PI4K also have different roles depending on cells. The inhibition of the two enzymes affects multiple cellular processes, including inhibition of clathrin mediated endocytosis [42], inhibition of vacuolar trafficking [43] and actin cytoskeleton organization [44]. The fact that both WM and LY294002 affected the kinetics of chloroplast responses more strongly than their amplitudes indicates that PI3K and PI4K might regulate yet other processes during the movement responses. In recent studies, Kong et al. [45] and Wan et al. [46] have shown BL induced phototropin internalization from plasma membrane to cytoplasm. However, neither the significance nor the regulators of this endocytic mechanism have been established.

**Figure 7:**
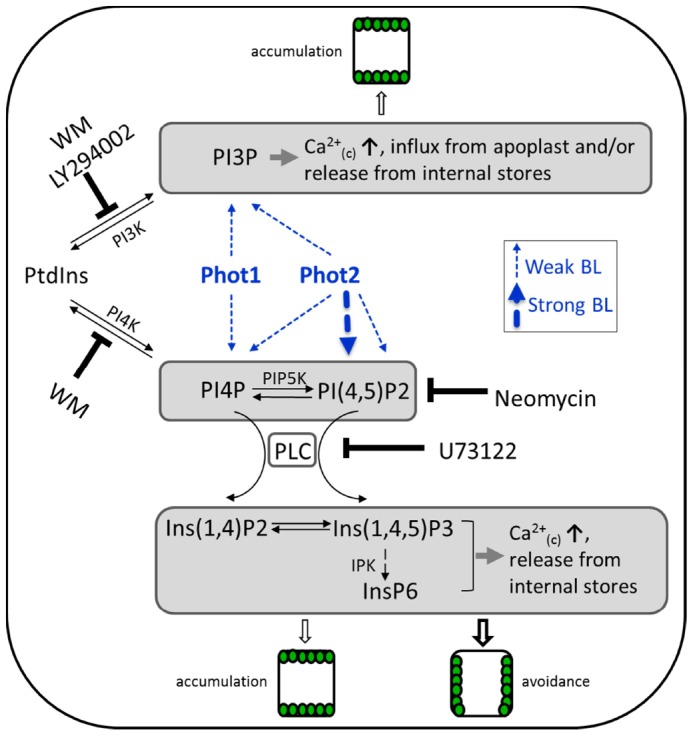
A model representing the postulated involvement of PIs and Ca^2+^ changes in the control of chloroplast responses in *Arabidopsis*. Phot1 induction by weak BL activates PI3K and PI4K, thereby producing PI3P and PI4P respectively. PI4P can be hydrolyzed by PLC generating water soluble Ins(1,4)P2. On the other hand, phot2 triggers activation of PI3K, PI4K and the PI(4,5)P2-PLC pathway upon weak BL induction, and only the PI(4,5)P2-PLC pathway upon strong BL. IPPs can be stepwise phosphorylated by inositolpolyphosphate multikinases, IPKs, to produce inositol hexaphosphate (InsP6), which has been also shown to be linked with Ca^2+^ mobilization [47]. One of the modes of action for PI3P, PI4P and PI(4,5)P2 during chloroplast relocations is calcium release in the cytoplasm. ↑ represents Ca^2+^ transient rise. PIP5K: phosphatidylinositol-4-phosphate 5-kinase and IPK: inositol polyphosphate kinase.

In summary, this study addresses a missing connection between phototropin activation and Ca^2+^ signaling and its relevance to the mechanism of chloroplast movements. The rapid turnover of PIs during phototropin-mediated signaling regulates chloroplast movements in *Arabidopsis*. PI(4,5)P2 hydrolysis via PLC and subsequent Ca^2+^ release are necessary for phot2-induced accumulation and avoidance responses. The other phosphorylated PI(s) produced from PI3K and PI4K are involved in regulating the phot1- and phot2-associated accumulation response partly through cytoplasmic Ca^2+^ signaling, and play only a minor role in the avoidance response. A working model of PI action consistent with our data is presented in **Fig. 7**. Several questions still remain to be answered in order to improve our understanding of phototropin signaling in plants. For example: 1) how do the phototropins switch between PI pathways at different fluence rates, 2) do cytosolic calcium changes determine the directionality of the chloroplast movements, 3) what other processes are regulated by PIs during movement responses.

## Supporting Information

Figure S1 **Basal levels of Ca^2+^_(c)_ before BL-induction.** Leaf discs reconstituted with coelenterazine were placed in the tube holder in luminometer and the basal level of Ca^2+^_(c)_ was recorded for 5 min. Leaves were treated with water, 10/50 µM WM, 200 µM LY294002, 10/25 µM U73122 or 10/25 µM U73343 60 min before the measurements.
(TIF)

Figure S2 **The actin cytoskeleton after neomycin treatment as visualized by alexa-fluor phalloidin staining.** After dark adaptation of the *Nicotiana tabacum* plants, lower epidermis was removed from the leaves. Subsequently, leaf tissue was cut into small pieces, infiltrated with 100 µM neomycin or with 10 mM PIPES buffer (pH 6.8) and incubated for 60 min in darkness before irradiation with BL. After the BL illumination (50 µmol m^−2 ^s^−1^, 45 min each), samples were infiltrated with actin stabilizing buffer (ASB: 50 mM PIPES, 10 mM EGTA, 5 mM MgSO_4_·7H2O) with 2% formaldehyde, 1% DMSO, 1 tablet of proteinase inhibitor cocktail and Na_2_-ATP. Samples were incubated for 2 h in darkness and then washed with ASB. Samples were then incubated for 1 h in staining solution (0.02 µM Alexa Fluor 488-Phalloidin and 1% DMSO, prepared in ASB). The condition of the tissue was checked by light microscopy after staining and confocal images were taken. Network of actin in dark-adapted mesophyll cells of tobacco after application of (**A**) 10 mM PIPES (pH 6.8) and (**B**) 100 µM neomycin. Reorganization of actin after 60 min exposure to strong BL (50 µmol m^−2^ s^−1^): (**C**) 10 mM PIPES and (**D**) 100 µM neomycin.
(TIF)
