# Supplementary figures and images for: Phosphoinositides Play Differential Roles in Regulating Phototropin1- and Phototropin2-Mediated Chloroplast Movements in Arabidopsis
Source: PLoS One. 2013 Feb 6;8(2):e55393. doi: 10.1371/journal.pone.0055393 (PMC3566141; doi:10.1371/journal.pone.0055393)

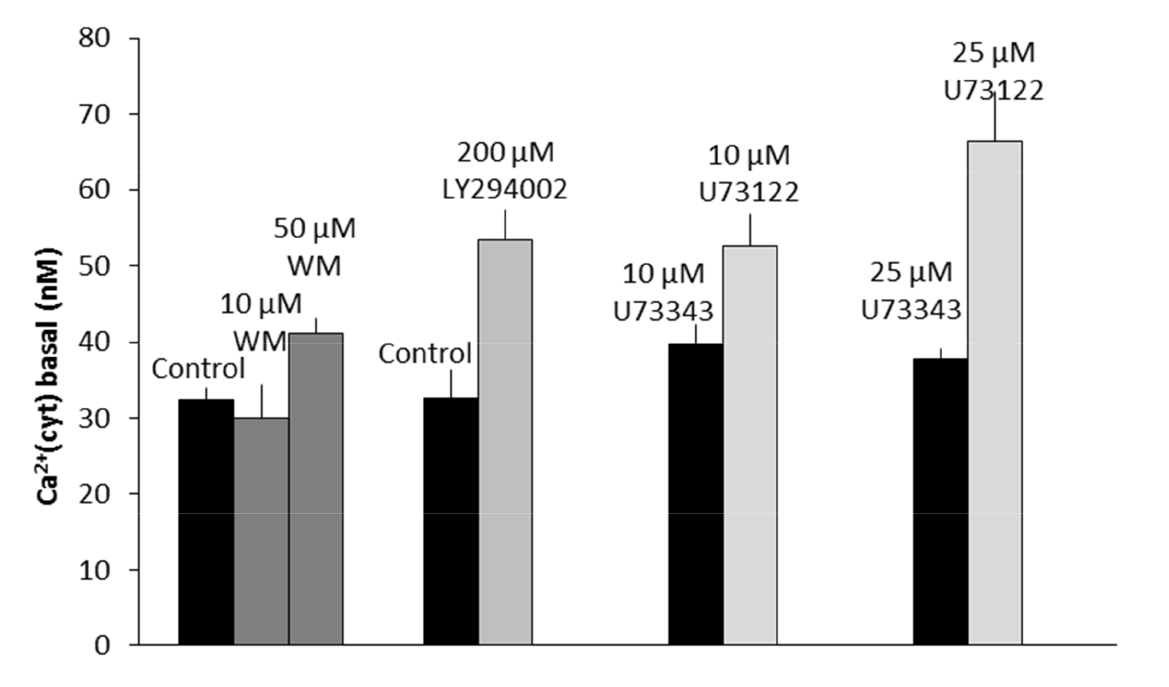

Supplement: Figure S1 — Basal levels of Ca2+(c) before BL-induction. Leaf discs reconstituted with coelenterazine were placed in the tube holder in luminometer and the basal level of Ca2+ (c) was recorded for 5 min. Leaves were treated with water, 10/50 µM WM, 200 µM LY294002, 10/25 µM U73122 or 10/25 µM U73343 60 min before the measurements. (TIF) [file pone.0055393.s001.tif]

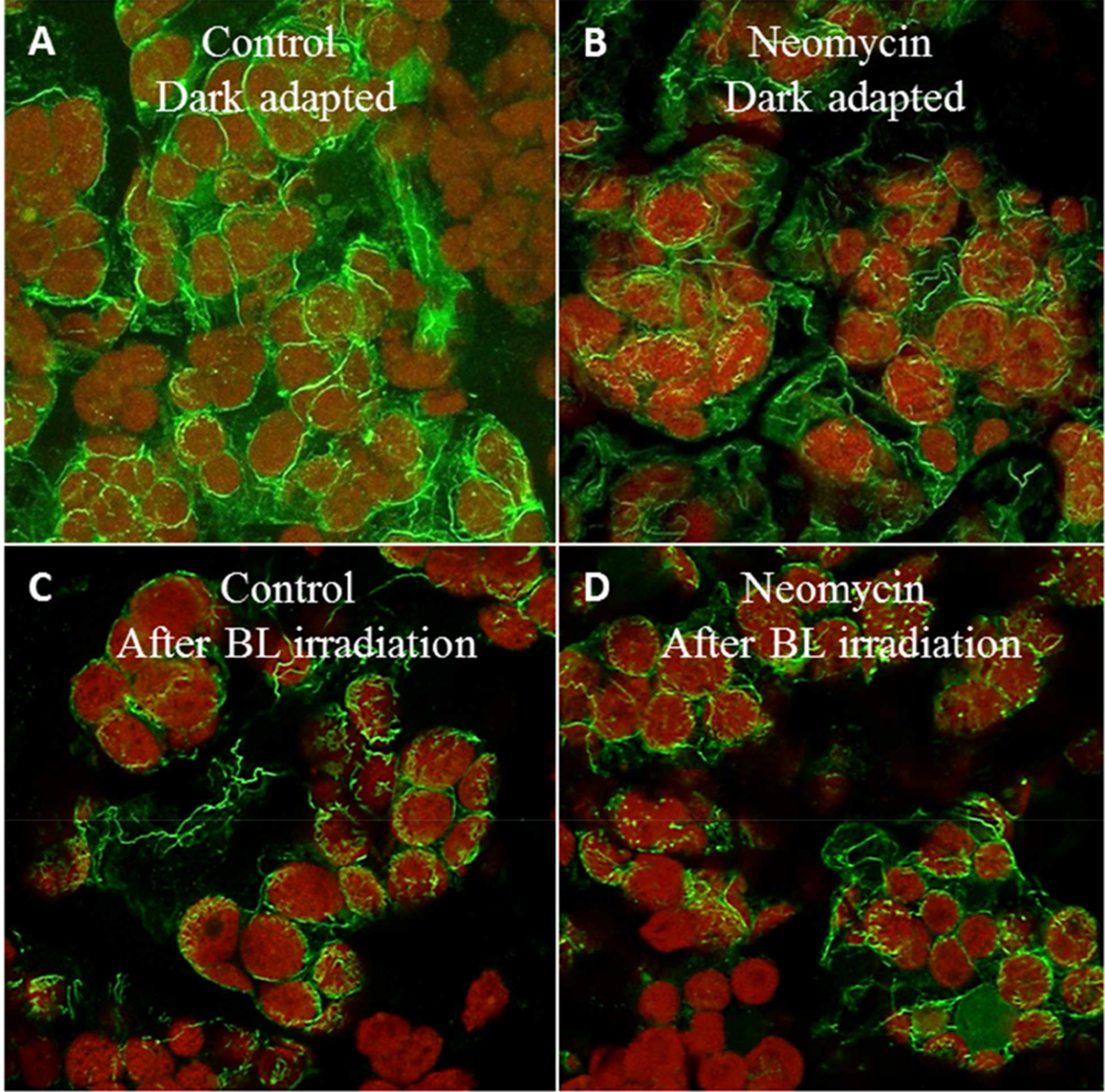

Supplement: Figure S2 — The actin cytoskeleton after neomycin treatment as visualized by alexa-fluor phalloidin staining. After dark adaptation of the Nicotiana tabacum plants, lower epidermis was removed from the leaves. Subsequently, leaf tissue was cut into small pieces, infiltrated with 100 µM neomycin or with 10 mM PIPES buffer (pH 6.8) and incubated for 60 min in darkness before irradiation with BL. After the BL illumination (50 µmol m−2 s−1, 45 min each), samples were infiltrated with actin stabilizing buffer (ASB: 50 mM PIPES, 10 mM EGTA, 5 mM MgSO4·7H2O) with 2% formaldehyde, 1% DMSO, 1 tablet of proteinase inhibitor cocktail and Na2-ATP. Samples were incubated for 2 h in darkness and then washed with ASB. Samples were then incubated for 1 h in staining solution (0.02 µM Alexa Fluor 488-Phalloidin and 1% DMSO, prepared in ASB). The condition of the tissue was checked by light microscopy after staining and confocal images were taken. Network of actin in dark-adapted mesophyll cells of tobacco after application of (A) 10 mM PIPES (pH 6.8) and (B) 100 µM neomycin. Reorganization of actin after 60 min exposure to strong BL (50 µmol m−2 s−1): (C) 10 mM PIPES and (D) 100 µM neomycin. (TIF) [file pone.0055393.s002.tif]
